# A collagen extraction and deuterium oxide stable isotope tracer method for the quantification of bone collagen synthesis rates *in vivo*

**DOI:** 10.14814/phy2.14799

**Published:** 2021-05-27

**Authors:** Rita Civil, Matthew S. Brook, Kirsty J. Elliott‐Sale, Lívia Santos, Ian Varley, Sanna Lensu, Heikki Kainulainen, Lauren G. Koch, Steven L. Britton, Daniel J. Wilkinson, Kenneth Smith, Craig Sale, Philip J. Atherton

**Affiliations:** ^1^ Musculoskeletal Physiology Research Group Sport Health and Performance Enhancement Research Centre School of Science and Technology Nottingham Trent University Nottingham UK; ^2^ Clinical, Metabolic and Molecular Physiology University of Nottingham Royal Derby Hospital Derby UK; ^3^ Department of Biology of Physical Activity University of Jyväskylä Jyväskylä Finland; ^4^ Department of Physiology and Pharmacology The University of Toledo Toledo OH USA; ^5^ Department of Anesthesiology University of Michigan Ann Arbor MI USA; ^6^ Department of Molecular and Integrative Physiology University of Michigan Ann Arbor MI USA

**Keywords:** bone turnover, collagen synthesis, deuterium oxide, GC‐*pyrolysis*‐IRMS, stable isotopes

## Abstract

The development of safe and practical strategies to prevent weakening of bone tissue is vital, yet attempts to achieve this have been hindered by a lack of understanding of the short‐term (days‐weeks) physiology of bone collagen turnover. To address this, we have developed a method to quantify bone collagen synthesis *in vivo*, using deuterium oxide (D_2_O) tracer incorporation techniques combined with gas chromatography pyrolysis isotope‐ratio mass spectrometry (GC‐*pyrolysis*‐IRMS). Forty‐six male and female rats from a selectively bred model ingested D_2_O for 3 weeks. Femur diaphyses (FEM), tibia proximal (T‐PRO), and distal (T‐DIS) epiphyses‐metaphyses and tibia mid‐shaft diaphyses (T‐MID) were obtained from all rats after necropsy. After demineralisation, collagen proteins were isolated and hydrolysed and collagen fractional synthetic rates (FSRs) determined by incorporation of deuterium into protein‐bound alanine via GC‐*pyrolysis*‐IRMS. The collagen FSR for the FEM (0.131 ± 0.078%/day; 95% CI [0.106–0.156]) was greater than the FSR at T‐MID (0.055 ± 0.049%/day; 95% CI [0.040–0.070]; *p* < 0.001). The T‐PRO site had the highest FSR (0.203 ± 0.123%/day; 95% CI [0.166–0.241]) and T‐DIS the lowest (0.027 ± 0.015%/day; 95% CI [0.022–0.031]). The three tibial sites exhibited different FSRs (*p* < 0.001). Herein, we have developed a sensitive method to quantify *in vivo* bone collagen synthesis and identified site‐specific rates of synthesis, which could be applicable to studies of human bone collagen turnover.

## Graphical abstract

We have developed a method to quantify bone collagen synthesis *in vivo*, using D_2_O in adult rodents combined with highly sensitive GC‐*pyrolysis*‐IRMS techniques. This method is ideally suited to the measurement of slow turnover proteins, such as collagen, and its high sensitivity will permit the application of D_2_O loading protocols that are well tolerated in humans (i.e., 150 ml + 50 ml/week^‐1^). Therefore, our D_2_O GC‐*pyrolysis*‐IRMS approach has great potential applicability to study human bone collagen turnover.

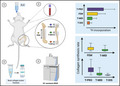

## 1 INTRODUCTION

Understanding bone remodelling in ageing and disease (e.g., osteoporosis), and developing strategies to maintain bone tissue are vital. Imaging techniques, such as dual‐energy X‐ray absorptiometry (DXA) and peripheral quantitative computed tomography, enable the measurement of the mineral compartment of bone. Changes in mineralised bone, however, can only be determined over a long period (e.g., months/years), and DXA‐derived bone mineral density only relates to about two‐thirds of the bone's strength (Ammann & Rizzoli, 2003). Other factors in the non‐mineral compartments of bone, are equally important (Burr, 2002). The extracellular matrix of bone, largely made up of collagen proteins, is vital in providing underlying strength to the bone (Burr, 2002).

Collagen is the most abundant protein in the human body, comprising ~30% of total body protein in humans (Smith & Rennie, 2007). In bone, 90% of the organic matrix is made up by type I collagen; and collagen (types I, II, III and V) is also an important component of tendon, skin, ligaments and muscle (Smith & Rennie, 2007). Human muscle collagen synthesis is slower than tendon collagen synthesis at rest (Miller et al., 2005; Smeets et al., 2019), although the question as to whether or not bone has a slower turnover than other musculoskeletal tissues remains controversial (Smeets et al., 2019; Smith & Rennie, 2007). The rate of bone collagen turnover is important in determining bone strength because it influences the pattern of mature/immature collagen crosslinking in bone, which is important for bone quality and strength (Bouxsein, 2005; Burr, 2002; Viguet‐Carrin et al., 2006). The mechanisms by which collagen turnover is altered in bone‐affecting diseases or in response to potentially favourable interventions (e.g., drugs, exercise, diet) to improve bone strength, is, however, poorly defined due to the lack of robust analytical approaches to its measurement.

Indirect measures of bone formation and resorption, known as bone (re)modelling markers, can be measured in the blood. Although these biomarkers are widely used for assessing short‐term changes in bone collagen turnover, they have yet to be validated against direct measures of bone collagen synthesis or breakdown (Babraj et al., 2005). Bone (re)modelling markers have some key limitations, including pre‐analytical (e.g., biological causes; sample collection, handling and storage requirements) and analytical (e.g., within and inter laboratory variation, assay reproducibility) variability (Hlaing & Compston, 2014; Lewiecki, 2010), as well as a lack of tissue and site specificity (Dolan et al., 2020; Smith & Rennie, 2007). As such, studies that have used bone biomarkers to measure collagen changes need to be interpreted carefully.

The direct incorporation of isotopically labelled tracers is generally regarded as the gold standard in determining fractional synthetic rates and can be performed on bone if a tissue sample can be collected (Wilkinson et al., 2017). Traditionally, amino acid isotope tracers, such as carbon (^13^C), deuterium (^2^H) or nitrogen (^15 ^N), have been used to measure *in vivo* synthesis of human musculoskeletal tissues, including bone (Babraj et al., 2005; Scrimgeour et al., 1993; Smeets et al., 2019). These amino acid tracers are, however, hindered by the heterogeneity of amino acid body pools (Brook et al., 2017; Wilkinson, 2016), and require preparation of high‐cost infusions and venous/arterial cannulation (Wilkinson et al., 2014). These studies are also restricted by time (generally <24 h), which makes it challenging to accurately measure very low rates of bone collagen synthesis. Furthermore, the use of variable stable isotope tracers and different bone protein fractions makes comparisons between studies difficult (Babraj et al., 2005; Scrimgeour et al., 1993; Smeets et al., 2019).

Using deuterium oxide (D_2_O or “heavy water”) as a stable isotope tracer can overcome some of these limitations. For instance, D_2_O can be easily ingested orally, with the deuterium becoming rapidly equilibrated within the body water and intracellular amino acid pools (Wilkinson et al., 2014). The potential to use protein‐bound alanine to quantify collagen synthesis offers a major advantage to detect low rates of tissue turnover such as in bone. First, up to four hydrogens are replaced by deuterium before free alanine is incorporated into newly made protein. This acts to amplify the amount of deuterium incorporated into the bound end product. Further, alanine has been robustly validated in the application of D_2_O methodologies (Wilkinson et al., 2014), with rapid transamination reactions meaning alanine enrichment is not easily perturbed overtime (Dufner et al., 2005). This allows D_2_O to be administered with minimal interference to an individual's normal daily activities, with enrichment in the precursor pool easily maintained over weeks and months (Wilkinson et al., 2017), making this tracer more suited to the measurement of slow turnover proteins, such as collagen.

Previous assessments of bone collagen synthesis rates using D_2_O have been made in rodents; however, these required high levels of ^2^H body water enrichment (~3%) and were performed in growing rats, where collagen synthesis rates are considerably higher (Busch et al., 2006; Do et al., 2006; Jeong et al., 2005). As such, methods using D_2_O to measure collagen synthesis rates in adult animals’ scenarios and potentially in humans, where collagen synthesis rates are considerably lower, are lacking. To address this, we have developed a method to quantify low levels of bone collagen synthesis *in vivo*, using lower levels (<1%) of ^2^H body water enrichment in adult rodents. Combining sensitive GC‐*pyrolysis*‐IRMS techniques, this method enables the measurement of slow turnover proteins such as collagen, with the potential to determine short term changes in bone collagen synthesis.

## 2 METHODS

### 2.1 Animals

All experiments were approved by the Animal Care and Use Committee of Southern Finland, license number ESAVI‐2010‐07989/Ym‐23, STH 534A (21.9.2010) and complements ESAVI/1968/04.10.03/2011, PH308A (30.3.2011) and ESAVI/722/04.10.07/2013, PH275A (1.3. 2013); and were conducted in accordance with the Guidelines of the European Community Council Directive 86/609/EEC. Bones were derived from 46 adult (9 ± 3 months) male (*n* = 22) and female (*n* = 24) rats , which were selectively bred for yielding low or high aerobic responses to exercise training (Koch et al., 2013). The background of the experimental animals is not relevant to the present method development and the bone was opportunistically harvested for this purpose as an addition to other independent investigations already being conducted.

Rats were single‐housed in air‐conditioned rooms at an ambient temperature of 21 ± 2°C and relative humidity at 50 ± 10%. Artificial lighting provided light cycles of 12:12‐h light‐total darkness. Commercially available pelleted rodent diet (R36; Labfor; Lantmän nen, Malmö, Sweden) and tap water (from the municipal water system of Jyväskylä, Finland) was available *ad libitum* throughout the study. The energy content of the feed was 1260 kJ/100 g (300.93 kcal/100 g). The feed contained 18.5% raw protein, 4.0% raw fat, 55.7% nitrogen‐free extracts, 3.5% fibre, 6.3% ash, and 12% water. Rats were divided into two groups of control or exercise trained, with samples collected from both groups for method development.

### 2.2 Deuterium enrichment

Rats received a gavage of 7.2 mL/kg 70% D_2_O, thereafter, animals were provided with free access to drinking water enriched with 2% (v/v) of D_2_O. Body water enrichment was determined from plasma and was used to calculate the average precursor enrichment. Blood samples were collected at necropsy (~5 mL) and plasma was separated by centrifugation and stored frozen until analysis. Body water enrichment was measured in plasma by incubating 100 µL of each sample with 2 µL of 10 M NaOH and 1 µL of acetone for 24 h at room temperature. Following incubation, the acetone was extracted into 200 µL of n‐heptane and 0.5 µL of the heptane phase was injected into the GC‐MS/MS for analysis. A standard curve of known D_2_O enrichment was run alongside the samples for calculation of enrichment.

### 2.3 Bone sample collection

Forty‐eight hours after the last training bout, animals were anesthetised with carbon dioxide and killed by cardiac puncture and thereafter immediately necropsied. Left femur and tibia bones were rapidly exposed, removed, and immediately frozen by complete immersion in liquid nitrogen and were kept at −80°C until analysis. We speculated that different anatomical bone sites might have different synthesis rates. As such, we obtained bone samples from the femur diaphysis (not site controlled) with pestle and mortar (FEM, 0.10 ± 0.03 g), and three different sites of the tibia using an electric hand saw (Dremel 3000 Rotary Tool, USA): tibial proximal epiphysis‐metaphysis (T‐PRO, 30 ± 0.08 g), the tibial mid‐shaft diaphysis (T‐MID, 9 ± 0.04 g), and the tibial distal epiphysis‐metaphysis (T‐DIS, 11 ± 0.02 g); each sample was ~20% of the total tibia length.

### 2.4 Isolation and derivatisation of bone collagen protein

Bone samples were transferred into 0.3–0.5 M HCl until samples were completely decalcified and appeared translucent and flexible. This process typically took 10–15 days, with the HCl solution being changed every 1–4 days. Following demineralisation, bone samples were transferred to 0.3 M NaOH in order to dissolve and remove the remaining bone marrow and soluble proteins, leaving the bone collagen proteins. The NaOH solution was changed ~3 times over 2–5 days with bouts of vortexing and centrifuging to help remove bone marrow particles. The remaining bone collagen proteins were hydrolysed to free amino acids by incubating in 0.1 M HCl in Dowex H^+^ resin slurry overnight at 110°C before being eluted from the resin with 2 M NH_4_OH and evaporated to dryness. Amino acids were then derivatised as their N‐methoxycarbonyl methyl esters. Dried samples were suspended in 60 µL of distilled water and 32 µL of methanol, and following vortex, 10 µL of pyridine and 8 µL of methyl chloroformate were added. Samples were vortexed for 30 s and left to react at room temperature for 5 min. The newly formed N‐methoxycarbonyl methyl ester amino acids were then extracted into 100 µL of chloroform. A molecular sieve was added to each sample for ~20 s before being transferred to a clean glass gas chromatography insert, removing any remaining water by size exclusion adsorption.

### 2.5 GC‐pyrolysis‐IRMS deuterated alanine analysis and calculation of fractional synthetic rates

Protein‐bound alanine enrichment was determined by gas chromatography pyrolysis isotope‐ratio mass spectrometry (GC‐*pyrolysis*‐IRMS) and body water enrichment by gas chromatography tandem mass spectrometry (GC‐MS/MS). Bone collagen fractional synthetic rates (FSR) were calculated from the incorporation of deuterium‐labelled alanine (corrected for the mean number of deuterium moieties incorporated per alanine [3.7] and the dilution from the total number of hydrogens in the derivative [i.e., 11]) into protein using the enrichment of body water as the surrogate precursor labelling over the 3‐week time period of D_2_O labelling. The equation used was:

$$\mathrm{FSR}=‐\ln\left[\frac{1‐\left(\frac{\mathrm{APEala}}{\mathrm{APEp}}\right)}{t}\right]$$

where APEala equals deuterium enrichment of protein‐bound alanine, APEp indicates mean precursor enrichment over the time period, and *t* represents time (3 weeks or 21 days) (Wilkinson et al., 2014).

### 2.6 Statistical analysis

Data from all rats were pooled and analysed together independently of the sex, phenotype and exercise for this study. Descriptive statistics were performed for all data sets to check for normal distribution (accepted if *p* > 0.05) using the Shapiro‐Wilk test. All data are presented as means ± 1SD. Differences between collagen FSR of the FEM and T‐MID samples were analysed by Wilcoxon matched pairs test. The Kruskal‐Wallis test was used to compare T‐PRO, T‐MID and T‐DIS samples. *Post hoc* analysis was performed using Dunn's multiple comparisons test to determine the differences between each of the tibial sites. All analyses were performed on GraphPad Prism 8 (La Jolla, CA, USA). The level of significance was set at *p* < 0.05 and 95% confidence intervals (95% CI) are presented for significant differences.

## 3 RESULTS

**FIGURE 1:**
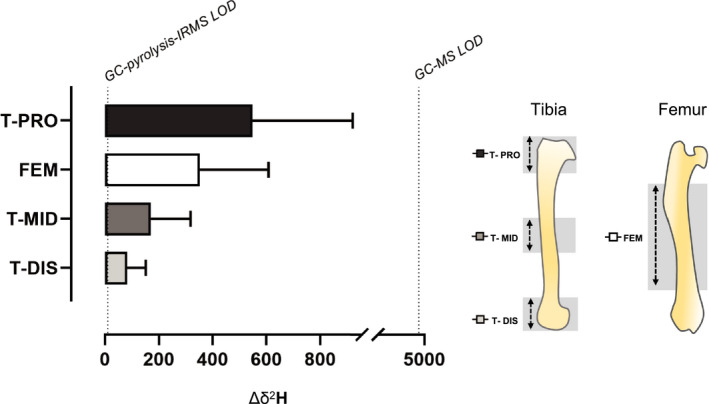
Change in bound deuterium enrichment (Δδ^2^H) across the tibia proximal (T‐PRO), mid‐shaft (T‐MID), distal (T‐DIS) and femur (FEM). GC‐*pyrolysis*‐IRMS limit of detection (LOD) shown as 10 δ^2^H and GC‐MS LOD shown as 4700 δ^2^H. Sampling areas of tibia and femur shown in highlighted in grey.

**FIGURE 2:**
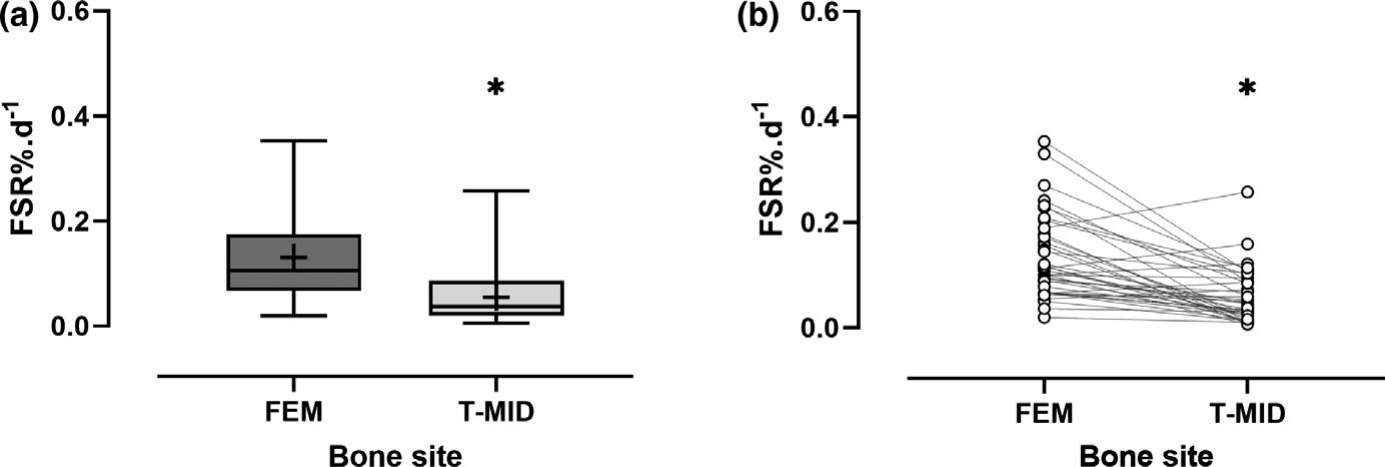
Collagen fractional synthetic rate (FSR) for the femur (FEM) and the mid‐shaft of the tibia (T‐MID). (a) Data represented as box plots, + represents mean. (b) Individual values. * Wilcoxon matched pairs test *p* < 0.001

**FIGURE 3:**
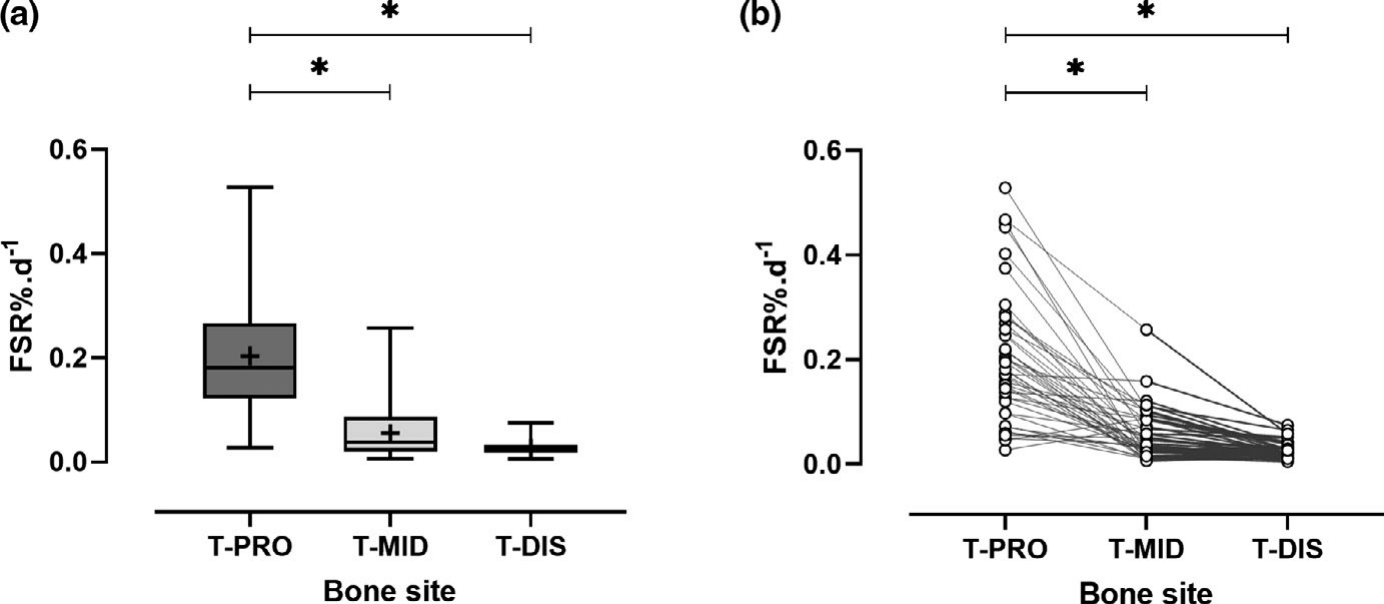
Collagen fractional synthetic rate (FSR) across the proximal (T‐PRO), mid‐shaft (T‐MID) and distal (T‐DIS) sites of the tibia. (a) Data represented as box plots, + represents the mean. (b) Individual values. * *Post hoc* Dunn's multiple comparisons test *p* < 0.001

The difference in rats’ body weight over a 7‐week period was ~6%. The average body water enrichment in rats was 0.685 ± 0.089 APE; whilst the average change in the deuterium labelling, expressed as delta per mil deuterium (δ^2^H), was FEM 352 ± 38 δ^2^H, T‐PRO 548 ± 45 δ^2^H, T‐MID 170 ± 21 δ^2^H and T‐DIS 83 ± 10 δ^2^H (Figure 1), with the higher the value reflecting the greater incorporation of labelled alanine. The calculated average collagen FSR for FEM (0.131 ± 0.078%/day; 95% CI [0.106–0.156]) were significantly greater than the FSR at T‐MID (0.055 ± 0.049%/day; 95% CI [0.040–0.070]; *p* < 0.001, Figure 2). The highest FSR was at the T‐PRO site (0.203 ± 0.123%/day; 95% CI [0.166–0.241]) and the lowest at the T‐DIS (0.027 ± 0.015%/day; 95% CI [0.022–0.031]). The three tibial sites had significantly different FSRs (*p* < 0.001, Figure 3). T‐PRO was significantly different from T‐MID (*p* < 0.001) and T‐DIS (*p* < 0.001), but the difference between T‐MID and T‐DIS was only approaching significance (*p* = 0.057).

## 4 DISCUSSION

We have developed a novel D_2_O stable isotope tracer method capable of detecting low levels of ^2^H incorporation and have tested this method for its ability to quantify a range of bone collagen synthesis rates *in vivo* over a 3‐week period in rodents. This method was able to detect differences in bone collagen synthesis between the femur and the tibia and differences in collagen FSR at different sites along the length of the same bone (tibia). Our measures of bone collagen FSR ranged between 0.005–0.529%/day, being ~30 fold lower than mean muscle protein synthesis rates measured in this cohort. Nonetheless, our data and others (Babraj et al., 2005; Smeets et al., 2019) suggest bone collagen has a greater turnover rate than previously suggested by semi‐quantitative estimates (3–25%/year) or calcium turnover (8–15%/year) (Smeets et al., 2019). Previous measures of murine bone collagen synthesis using D_2_O have showed active synthesis (Do et al., 2006; Jeong et al., 2005), with one study reporting rates of ~17%/week in young growing mice (Busch et al., 2006).

Tibial samples were obtained from the mid‐shaft diaphysis site (corrected by the length of each rat's bone). In addition, samples from proximal and distal epiphyses‐metaphyses were obtained, in order to investigate collagen FSRs across bone regions that have different compositions of trabecular and cortical bone. Diaphysis synthesis rates in the femur (not site‐controlled) and tibia (mid‐shaft) were significantly different. The synthesis rate was faster at the proximal tibial site than at both the mid‐shaft and distal sites. The differences between collagen FSR across bone sites reported herein highlight the potential limitations in the utility of bone (re)modelling biomarkers that estimate whole body bone turnover. In fact, previous work in humans has pointed to the incongruities between the PINP bone formation biomarker and changes in bone (Babraj et al., 2005) and tendon (Miller et al., 2007) synthesis. Further, our results highlight the importance of controlling and reporting the bone site used for analysis in future studies.

Such differences between synthesis rates among bone sites may be due to variability of strain distribution and magnitude across bone surfaces when physical loading is applied, producing an osteogenic effect (i.e., stimulation of bone formation). For example, similar bone‐site differences in 19‐week‐old mice were shown using μCT and histomorphometry analyses (Sugiyama et al., 2010). After receiving *in vivo* artificial loading for 2 weeks, murine tibia showed greater changes and new bone formation in the proximal and mid‐shaft sites compared to the distal site (Sugiyama et al., 2010). The heterogeneity of bone may well be important when considering how mechanical loading affects trabecular and cortical bone since they appear to respond different to loading (Yang et al., 2017). Trabecular bone, compared to cortical bone, has shown a higher response to changes in the loading environment in mice vertebrae (Lambers et al., 2011) and tibia (Fritton et al., 2005). A different study showed that cortical and trabecular bone expressed different genes at baseline and in response to *in vivo* mechanical loading (Kelly et al., 2016), suggesting that the cellular mechanisms of the mechanical loading responses in trabecular and cortical bone are different. This could explain the higher collagen FSRs at the proximal site of the rat tibia (composed of more trabecular bone), compared to the mid‐shaft and distal tibia (composed of more cortical bone) shown herein.

Another important factor influencing an osteogenic response is the muscle contractile forces exerted upon the skeleton during movement (Hart et al., 2017). The direct insertion of healthy and active muscle tissue onto the bone periosteum promotes localised bone formation without mechanical stimulation (Hart et al., 2017). Herein, we showed greater collagen synthesis at the proximal site of the tibia, with major muscles being adjacent to this region of the knee. We are confident that the differences in collagen FSRs shown across different bone sites were not due to contamination with protein or amino acids from bone marrow or connective tissue, since care was taken during sample preparation to ensure bone samples were clean. Bone marrow and alkali soluble protein was thoroughly removed with 0.3 M NaOH and the remaining connective tissue was manually removed with a sharp scalpel during the demineralisation process.

Additionally, the potential presence of periosteum and growth plate in the tibial epiphyses (proximal and distal) may also have affected our measurements of bone collagen synthesis. Wilsman et al. suggested that the tibial proximal growth plate has a greater growth rate compared the tibial distal growth plate in 2–4‐week‐old rats (Wilsman et al., 1996, 2008). We showed a higher collagen synthesis rate in the proximal tibia than in the distal tibia and mid‐shaft, although the collagen synthesis rate in the distal tibia had a slower synthesis rate than the mid‐shaft site (without a growth plate). Whilst rats used in our study were 9 months old at the start of the study, histological evidence suggests that tibial proximal growth plates are still active (areas of resting cells, cell proliferation, cell maturation and lacunar hypertrophy) and cartilage is still present in up to 25 month old rats, despite bony bridging being complete and without longitudinal bone growth (Martin et al., 2003). As such, it is possible that there were some elements of the growth plates present in the proximal and distal tibial sites measures, although we cannot determine the exact extent to which this might have affected our interpretation of their collagen synthesis rates. However, this issue will likely be minimised in human studies, where the control for the bone site during sampling can be made more easily in larger bones and the growth plates close in late puberty (Kember & Sissons, 1976; Shim, 2015). Future application of this method in interventional studies will provide further validity of the method and its sensitivity.

To date, no studies have used D_2_O as a direct incorporation tracer technique to determine human bone collagen synthesis. The use of D_2_O has many advantages for determining bone synthesis compared to traditional amino acid tracer approaches, where changes in bone synthesis can only be captured in a short timeframe (i.e., over hours). D_2_O labelling with continued oral ingestion of heavy water can safely maintain body water enrichment for days, weeks, or months (Wilkinson et al., 2017). This is especially important for slow turnover proteins, such as collagen, which may need longer periods of labelling for longer term interventional studies looking at changes in bone synthesis. Our bone collagen extraction and D_2_O GC‐*pyrolysis*‐IRMS method offers a highly sensitive technique for quantifying small changes in δ^2^H and therefore bone collagen synthesis *in vivo*. Despite very low collagen synthesis rates, this method will ultimately permit measures of bone collagen synthesis in humans using well tolerated D_2_O loading protocols (i.e., 150 + 50 mL/week^−1^). As such, there is great future applicability to human investigations, which are crucial in determining differences in bone turnover between age, sex, health and disease, and responses to interventions, such as exercise, diet and drugs.

## AUTHOR CONTRIBUTION

Conceptualisation: RC, MSB, KJE, LS, IV, DJW, KS, CS, and PJA. Methodology: RC, MSB, HK, SL, LGK, SLB, DJW, KS, CS, and PJA. Formal analysis: RC and MSB. Investigation: RC, MSB, HK, SL, LGK, and SLB. Data curation: RC, MSB, CS and PJA. Resources: MSB, HK, SL, LGK, SLB, DJW, KS, CS, and PJA. Writing original draft: RC. Writing review and editing: RC, MSB, KJE, LS, IV, HK, SL, LGK, SLB, DJW, KS, CS, and PJA. Visualisation: RC, MSB, CS and PJA. Supervision: MSB, KJE, LS, IV, DJW, KS, CS and PJA. Funding acquisition: CS and PJA.

## References

[phy214799-bib-0001] Ammann, P. , & Rizzoli, R. (2003). Bone strength and its determinants. Osteoporosis International, 14(Suppl 3), 13–18. 10.1007/s00198-002-1345-4. 12730800

[phy214799-bib-0002] Babraj, J. A. , Smith, K. , Cuthbertson, D. J. R. , Rickhuss, P. , Dorling, J. S. , & Rennie, M. J. (2005). Human bone collagen synthesis is a rapid, nutritionally modulated process. Journal of Bone and Mineral Research, 20, 930–937. 10.1359/JBMR.050201. 15883632

[phy214799-bib-0003] Bouxsein, M. L. (2005). Determinants of skeletal fragility. Best Practice & Research Clinical Rheumatology, 19, 897–911. 10.1016/j.berh.2005.07.004. 16301186

[phy214799-bib-0004] Brook, M. S. , Wilkinson, D. J. , Atherton, P. J. , & Smith, K. Recent developments in deuterium oxide tracer approaches to measure rates of substrate turnover: implications for protein, lipid, and nucleic acid research. Current Opinion in Clinical Nutrition and Metabolic Care, 20(5), 375–381. 28650854 10.1097/MCO.0000000000000392

[phy214799-bib-0005] Burr, D. B. (2002). The contribution of the organic matrix to bone's material properties. Bone, 31, 8–11. 10.1016/S8756-3282(02)00815-3. 12110405

[phy214799-bib-0006] Busch, R. , Kim, Y. K. , Neese, R. A. , Schade‐Serin, V. , Collins, M. , Awada, M. , Gardner, J. L. , Beysen, C. , Marino, M. E. , Misell, L. M. , & Hellerstein, M. K. (2006). Measurement of protein turnover rates by heavy water labeling of nonessential amino acids. Biochimica Et Biophysica Acta (BBA) ‐ General Subjects, 1760, 730–744. 10.1016/j.bbagen.2005.12.023. 16567052

[phy214799-bib-0007] Do, S. H. , Il, J. W. , Jeong, D. H. , Ki, M. R. , Lee, I. S. , Kwak, D. M. , Kim, T. H. , Kim, Y. K. , Kim, S. B. , & Jeong, K. S. (2006). Alcohol‐induced bone degradation and its early detection in the alcohol‐fed castrated rats. Molecular and Cellular Biochemistry, 282, 45–52. 10.1007/s11010-006-1155-7. 16317511

[phy214799-bib-0008] Dolan, E. , Varley, I. , Ackerman, K. E. , Pereira, R. M. R. , Elliott‐Sale, K. J. , & Sale, C. (2020). The bone metabolic response to exercise and nutrition. Exercise and Sport Sciences Reviews, 48(2), 49–58. 10.1249/JES.0000000000000215. 31913188

[phy214799-bib-0009] Dufner, D. A. , Bederman, I. R. , Brunengraber, D. Z. , Rachdaoui, N. , Ismail‐Beigi, F. , Siegfried, B. A. , Kimball, S. R. , & Previs, S. F. (2005). Using 2H2O to study the influence of feeding on protein synthesis: Effect of isotope equilibration in vivo vs. in cell culture. American Journal of Physiology. Endocrinology and Metabolism, 288, 1277–1283. 10.1152/ajpendo.00580.2004. 15671077

[phy214799-bib-0010] Fritton, J. C. , Myers, E. R. , Wright, T. M. , & Van Der Meulen, M. C. H. (2005). Loading induces site‐specific increases in mineral content assessed by microcomputed tomography of the mouse tibia. Bone, 36, 1030–1038. 10.1016/j.bone.2005.02.013. 15878316

[phy214799-bib-0011] Hart, N. H. , Nimphius, S. , Rantalainen, T. , Ireland, A. , Siafarikas, A. , & Newton, R. U. (2017). Mechanical basis of bone strength: Influence of bone material, bone structure and muscle action. Journal of Musculoskeletal and Neuronal Interactions, 17, 114–139. 28860414 PMC5601257

[phy214799-bib-0012] Hlaing, T. T. , & Compston, J. E. (2014). Biochemical markers of bone turnover ‐ uses and limitations. Annals of Clinical Biochemistry, 51, 189–202. 10.1177/0004563213515190. 24399365

[phy214799-bib-0013] Jeong, K. S. , Lee, J. , Jeong, W. , Noh, D. H. , Do, S. H. , & Kim, Y. K. (2005). Measurement of estrogen effect on bone turnover by 2H2O labeling. Calcified Tissue International, 76, 365–370. 10.1007/s00223-004-1103-z. 15742235

[phy214799-bib-0014] Kelly, N. H. , Schimenti, J. C. , Ross, F. P. , & van der Meulen, M. C. H. (2016). Transcriptional profiling of cortical versus cancellous bone from mechanically‐loaded murine tibiae reveals differential gene expression. Bone, 86, 22–29. 10.1016/j.bone.2016.02.007. 26876048 PMC4833881

[phy214799-bib-0015] Kember, N. F. , & Sissons, H. A. (1976). Quantitative histology of the human growth plate. The Journal of Bone and Joint Surgery. British Volume, 58, 426–435. 10.1302/0301-620x.58b4.1018028. 1018028

[phy214799-bib-0016] Koch, L. G. , Pollott, G. E. , & Britton, S. L. (2013). Selectively bred rat model system for low and high response to exercise training. Physiological Genomics, 45, 606–614. 10.1152/physiolgenomics.00021.2013. 23715262 PMC3727016

[phy214799-bib-0017] Lambers, F. M. , Schulte, F. A. , Kuhn, G. , Webster, D. J. , & Müller, R. (2011). Mouse tail vertebrae adapt to cyclic mechanical loading by increasing bone formation rate and decreasing bone resorption rate as shown by time‐lapsed in vivo imaging of dynamic bone morphometry. Bone, 49, 1340–1350. 10.1016/j.bone.2011.08.035. 21964411

[phy214799-bib-0018] Lewiecki, E. M. (2010). Benefits and limitations of bone mineral density and bone turnover markers to monitor patients treated for osteoporosis. Current Osteoporosis Reports, 8, 15–22. 10.1007/s11914-010-0004-5. 20425086

[phy214799-bib-0019] Martin, E. A. , Ritman, E. L. , & Turner, R. T. (2003). Time course of epiphyseal growth plate fusion in rat tibiae. Bone, 32, 261–267. 10.1016/S8756-3282(02)00983-3. 12667553

[phy214799-bib-0020] Miller, B. F. , Hansen, M. , Olesen, J. L. , Schwarz, P. , Babraj, J. A. , Smith, K. , Rennie, M. J. , & Kjaer, M. (2007). Tendon collagen synthesis at rest and after exercise in women. Journal of Applied Physiology, 102, 541–546. 10.1152/japplphysiol.00797.2006. 16990502

[phy214799-bib-0021] Miller, B. F. , Olesen, J. L. , Hansen, M. , Døssing, S. , Crameri, R. M. , Welling, R. J. , Langberg, H. , Flyvbjerg, A. , Kjaer, M. , Babraj, J. A. , Smith, K. , & Rennie, M. J. (2005). Coordinated collagen and muscle protein synthesis in human patella tendon and quadriceps muscle after exercise. Journal of Physiology, 567, 1021–1033. 10.1113/jphysiol.2005.093690. PMC1474228 16002437

[phy214799-bib-0022] Scrimgeour, C. M. , Downie, S. , Rickuss, P. K. , & Rennie, M. (1993). Collagen synthesis in human bone measured using stable isotope‐labelled alanine and proline. Proceedings of the Nutrition Society London, 52, 258A.

[phy214799-bib-0023] Shim, K. S. (2015). Pubertal growth and epiphyseal fusion. Annals of Pediatric Endocrinology & Metabolism, 20, 8. 10.6065/apem.2015.20.1.8. 25883921 PMC4397276

[phy214799-bib-0024] Smeets, J. S. J. , Horstman, A. M. H. , Vles, G. F. , Emans, P. J. , Goessens, J. P. B. , Gijsen, A. P. , van Kranenburg, J. M. X. , & van Loon, L. J. C. (2019). Protein synthesis rates of muscle, tendon, ligament, cartilage, and bone tissue in vivo in humans. PLoS One, 14, 1–17. 10.1371/journal.pone.0224745. PMC6837426 31697717

[phy214799-bib-0025] Smith, K. , & Rennie, M. J. (2007). New approaches and recent results concerning human‐tissue collagen synthesis. Current Opinion in Clinical Nutrition and Metabolic Care, 10, 582–590. 10.1097/MCO.0b013e328285d858. 17693741

[phy214799-bib-0026] Sugiyama, T. , Price, J. S. , & Lanyon, L. E. (2010). Functional adaptation to mechanical loading in both cortical and cancellous bone is controlled locally and is confined to the loaded bones. Bone, 46, 314–321. 10.1016/j.bone.2009.08.054. 19733269 PMC2825292

[phy214799-bib-0027] Viguet‐Carrin, S. , Garnero, P. , & Delmas, P. D. (2006). The role of collagen in bone strength. Osteoporosis International, 17, 319–336. 10.1007/s00198-005-2035-9. 16341622

[phy214799-bib-0028] Wilkinson, D. D. J. (2016). Historical and contemporary stable isotope tracer approaches to studying mammalian protein metabolism. Mass Spectrometry Reviews, 47, 987–992. 10.1002/mas.21507 PMC5763415 27182900

[phy214799-bib-0029] Wilkinson, D. J. , Brook, M. S. , Smith, K. , & Atherton, P. J. (2017). Stable isotope tracers and exercise physiology: Past, present and future. Journal of Physiology, 595, 2873–2882. 10.1113/JP272277. PMC5407962 27610950

[phy214799-bib-0030] Wilkinson, D. J. , Franchi, M. V. , Brook, M. S. , Narici, M. V. , Williams, J. P. , Mitchell, W. K. , Szewczyk, N. J. , Greenhaff, P. L. , Atherton, P. J. , & Smith, K. (2014). A validation of the application of D2O stable isotope tracer techniques for monitoring day‐to‐day changes in muscle protein subfraction synthesis in humans. American Journal of Physiology ‐. Endocrinology and Metabolism, 306, 571–579. 10.1152/ajpendo.00650.2013. PMC3948971 24381002

[phy214799-bib-0031] Wilsman, N. J. , Bernardini, E. S. , Leiferman, E. , Noonan, K. , & Farnum, C. E. (2008). Age and pattern of the onset of differential growth among growth plates in rats. Journal of Orthopaedic Research, 26, 1457–1465. 10.1002/jor.20547. 18404738 PMC2954232

[phy214799-bib-0032] Wilsman, N. J. , Farnum, C. E. , Green, E. M. , Lieferman, E. M. , & Clayton, M. K. (1996). Cell cycle analysis of proliferative zone chondrocytes in growth plates elongating at different rates. Journal of Orthopaedic Research, 14, 562–572. 10.1002/jor.1100140410. 8764865

[phy214799-bib-0033] Yang, H. , Embry, R. E. , & Main, R. P. (2017). Effects of loading duration and short rest insertion on cancellous and cortical bone adaptation in the mouse tibia. PLoS One, 12, 1–20. 10.1371/journal.pone.0169519. PMC5226737 28076363

